# *Neisseria gonorrhoeae* induces local secretion of IL-10 at the human cervix to promote colonization

**DOI:** 10.1172/JCI183331

**Published:** 2024-11-14

**Authors:** Yiwei Dai, Vonetta L. Edwards, Qian Yu, Hervé Tettelin, Daniel C. Stein, Wenxia Song

**Affiliations:** 1 Department of Cell Biology and Molecular Genetics, University of Maryland, College Park, Maryland, USA.; 2 Department of Microbiology and Immunology, Institute for Genome Sciences, University of Maryland School of Medicine, Baltimore, Maryland, USA.

**Keywords:** Infectious disease, Bacterial infections, Cytokines

## Abstract

Gonorrhea, caused by the human-restricted pathogen *Neisseria gonorrhoeae*, is a commonly reported sexually transmitted infection. Since most infections in women are asymptomatic, the true number of infections is likely much higher than reported. How gonococci (GC) colonize women’s cervixes without triggering symptoms remains elusive. Using a human cervical tissue explant model, we found that GC inoculation increased the local secretion of both proinflammatory (IL-1β and TNF-α) and antiinflammatory (IL-10) cytokines during the first 24 hours of infection. Cytokine induction required GC expression of Opa isoforms that bind the host receptors carcinoembryonic antigen-related cell adhesion molecules (CEACAMs). GC inoculation induced NF-κB activation in both cervical epithelial and subepithelial cells. However, inhibition of NF-κB activation, which reduced GC-induced IL-1β and TNF-α, did not affect GC colonization. Neutralizing IL-10 or blocking IL-10 receptors by antibodies reduced GC colonization by increasing epithelial shedding and epithelial cell-cell junction disassembly. Inhibition of the CEACAM downstream signaling molecule SHP1/2, which reduced GC colonization and increased epithelial shedding, decreased GC-induced IL-10 secretion. These results show that GC induce local secretion of IL-10, a potent antiinflammatory cytokine, at the cervix by engaging the host CEACAMs to prevent GC-colonizing epithelial cells from shedding, providing a potential mechanism for GC asymptomatic colonization in women.

## Abstract 2

*Neisseria gonorrhoeae* suppresses immune detection and promotes bacterial colonization at the human cervix by inducing the local secretion of the potent anti-inflammatory cytokine IL-10.

## Graphical abstract

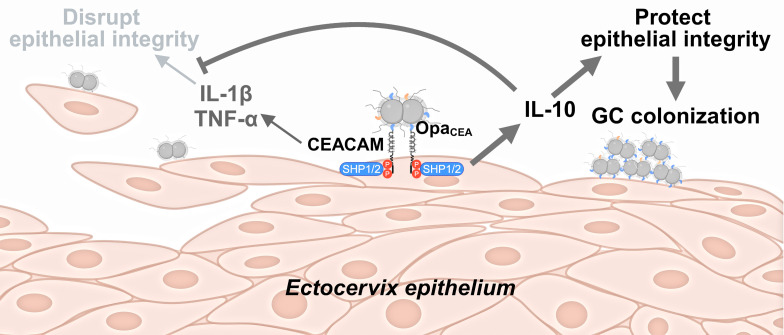

## Introduction

Gonorrhea, caused by the Gram-negative bacterium *Neisseria gonorrhoeae*, is the second most common sexually transmitted infection (1). In women, most infections are asymptomatic or subclinical; therefore, the true number of infections is likely far higher than reported (2, 3). Gonococci (GC) infect humans exclusively, primarily targeting male and female urethras and the female cervix. GC can ascend from the vagina to the upper female reproductive tract (FRT) through the cervix, leading to severe clinical consequences, including pelvic inflammatory disease and infertility (4, 5). GC can colonize the lower part of the FRT asymptomatically for weeks and months (6, 7), allowing the bacteria to silently spread among sex partners and delaying the treatment until the infection permanently damages the FRT. Despite the clinical importance of asymptomatic infection, how GC colonize the human cervix without inducing symptoms or eliciting immune responses remains elusive. The primary obstacles in research of asymptomatic GC infection are the difficulties in clinically identifying and tracking asymptomatic carriers from the beginning of an infection and a lack of models that mimic asymptomatic infection in humans.

GC initiate infection at the FRT by colonizing the luminal epithelial cells of the cervix (8, 9) and can invade into epithelial cells (10, 11) and/or transmigrate across columnar epithelial cells (12, 13). GC use pili to initiate the interaction with epithelial cells, making them essential for GC colonization at the cervical epithelia (14–16). Mechanistically, pilus retraction brings GC close to the luminal surface of epithelial cells (15, 17), allowing for the interaction of GC surface molecules, such as opacity-associated proteins (Opa), with their human-specific host receptors, such as carcinoembryonic antigen-related cell adhesion molecules (CEACAMs) and heparan sulfate proteoglycans (HSPGs) (18, 19). We have previously established a human cervical tissue explant model and shown that this model recapitulates GC infection in vivo (16). GC preferentially colonize the ectocervix and transformation zone but selectively penetrate into the subepithelia of the transformation zone and endocervix of tissue explants, mimicking observations of patients’ biopsies (8, 9, 16). Opas binding to CEACAMs (Opa_CEA_) promote GC colonization and inhibit GC penetration at the human cervix by inhibiting GC-induced epithelial cell-cell junction disruption and epithelial shedding (13, 16, 20, 21). The inhibitory effects of Opa_CEA_ on GC penetration depend on the immunoreceptor tyrosine-based inhibitory motif (ITIM) in the cytoplasmic tails of CEACAM1 and its downstream signaling molecule SH2-containing protein tyrosine phosphatase 1/2 (SHP1/2) (16). GC-induced epithelial cell shedding and the inhibitory effects of Opa_CEA_ have also been observed in the cervixes of vaginally infected mice that express human CEACAM5 and in human epithelial cell lines (20, 21). The fact that most of the 11 isoforms of Opa proteins bind to CEACAMs (22, 23) suggests that Opa_CEA_-promoted colonization but not penetration enables GC survival on the human cervix.

The local cytokine response induced by microbial pathogens is one of the earliest warning signals for the immune system (24, 25). Inflammatory chemokines and cytokines can chemoattract and activate resident and circulating immune cells to clear the infection, with neutrophil infiltration being the main clinical feature of symptomatic GC infection (26). In contrast, a lack of a robust inflammatory cytokine response or an antiinflammatory cytokine-dominated response can prohibit neutrophil infiltration (27). How GC escape or suppress local inflammatory responses to colonize women’s cervix silently remains a critically unanswered question. The relationship between GC asymptomatic infection and the initial local cytokine response of the cervix is unknown. Both inflammatory cytokines, such as IL-1β, TNF-α, and IL-17, and antiinflammatory cytokines, such as IL-10 and TGF-β, have been detected in the cervical mucus of uninfected women (28–30). Elevation, reduction, and no change of both proinflammatory and antiinflammatory cytokines have all been reported previously in the cervical secretion of patients with GC infection, compared with uninfected controls (28, 31–34). This broad spectrum of data probably resulted from variations in sample collecting methods, sample collecting times relative to the menstrual cycle and GC infection course, and whether the infection was symptomatic or asymptomatic. In vitro studies have shown that GC induce the production of the antiinflammatory cytokines IL-10 by human monocyte-derived dendritic cells (35), mouse bone marrow–derived dendritic cells (36), human monocyte-derived macrophages (37), human CD4^+^ T cells (38), human and mouse peripheral blood mononuclear cells (39, 40), mouse genital tract tissue explants (40), and cells from mouse iliac lymph nodes (40). As IL-10 is a master regulator of immunity and functions to protect hosts from overexuberant responses (41–43), these data suggest that inducing an antiinflammatory cytokine response is a potential mechanism for GC to evade the host adaptive immunity when GC can enter tissues and directly interact with immune cells. However, the kind of cytokine responses colonizing GC can induce initially and locally at the human cervix, and how induced cytokines impact immune detection are unknown.

In addition to activating inflammatory and antiinflammatory responses, cytokines have been shown to regulate the barrier function and the homeostasis of the epithelium (44–46), potentially influencing the infection processes. The proinflammatory cytokines IL-1β and TNF-α, which are associated with inflammatory bowel diseases, disrupt epithelial cell-cell junctions by increasing the expression and activation levels of myosin light chain kinase (MLCK) (47–49). TNF-α can also damage the epithelial barrier by inducing epithelial apoptosis, leading to shedding (50). In contrast, the antiinflammatory cytokine IL-10 activates epithelial cell proliferation and wound repair, strengthening their epithelial cell barrier function (42, 43, 51, 52). The deficiency of IL-10 or IL-10 receptor (IL-10R) leads to human inflammatory bowel diseases (53, 54). However, the pleiotropic cytokine TGF-β can strengthen (such as in the intestine) (55) or weaken (such as in the uterus) (56) epithelial cell-cell junctions depending on its anatomic location. The cytokine environment of the human cervix and its role in GC infection have not been well studied.

This study examined the relationship between locally secreted cytokines and GC infection when the cervix is initially exposed to GC, using our human cervical tissue explant model. The explant model preserves the heterogeneous cervical epithelia and subepithelial cells and mimics in vivo GC infection with GC predominately colonizing the cervical epithelium (8, 9, 13, 16). Furthermore, it enables us to examine the earliest local cytokine response to GC colonization without the influence of blood and lymphoid circulation. This study focused on cytokines detected in female secretions and known to regulate epithelial integrity. Our results show that GC inoculation increased the secretion of the proinflammatory cytokines TNF-α and IL-1β by activating NF-κB and the secretion of the antiinflammatory cytokine IL-10 by engaging the host receptor CEACAMs by Opa and activating CEACAM1 downstream signaling molecule SHP1/2. GC-induced proinflammatory cytokines TNF-α and IL-1β did not significantly affect GC colonization at the ectocervix. However, GC-induced antiinflammatory cytokine IL-10 enhanced GC colonization at the ectocervix by inhibiting GC-induced epithelial cell-cell junction disruption and the shedding of GC-associated cervical epithelial cells.

## Results

### GC inoculation increases the local secretion levels of both proinflammatory and antiinflammatory cytokines in the human cervix.

**Figure 1:**
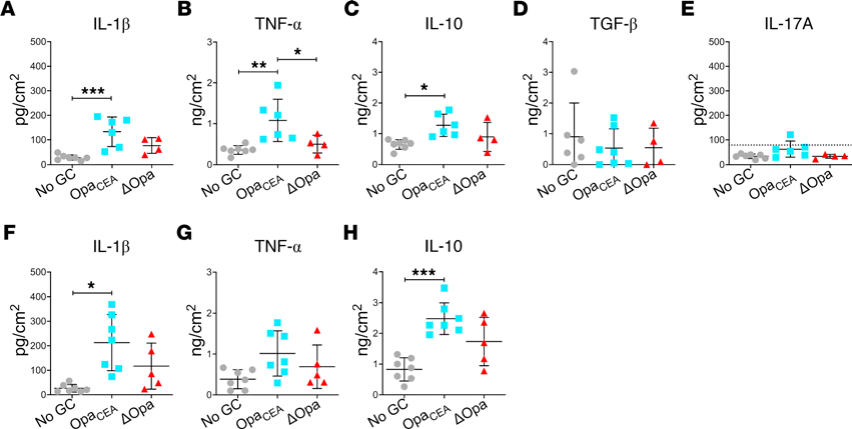
GC inoculation increases the secretion of both proinflammatory and antiinflammatory cytokines by human cervical tissue explants. Human cervical tissue explants with the ecto- to endocervix (**A**–**E**) or with the ectocervix alone (**F**–**H**) were incubated without (No GC) or with MS11 GC strains expressing no *opa* genes (ΔOpa) or a nonphase variable Opa_52_ that binds to CEACAMs (Opa_CEA_) at a MOI of 10 for 24 hours at 37^o^C. Supernatants of cervical tissue explant media were collected. The average concentrations (± SD) of IL-1β (**A** and **F**), TNF-α (**B** and **G**), IL-10 (**C** and **H**), TGF-β (**D**), and IL-17A (**E**, dashed line, detection level) were measured by Luminex Magpix (IL-1β, TNF-α, IL-10, and IL-17A) or ELISA (TGF-β), normalized to the luminal surface areas of tissue explants and supernatant volumes. Data points represent cervical tissues from individuals. The data were generated from 3 to 7 cervixes. *P < 0.05, **P < 0.01, ***P < 0.001, by 1-way ANOVA.

To determine the initial, local cytokine response of the cervix to GC infection, human cervical tissue explants (ecto- to endocervix) from the same donor were incubated without (no GC) or with MS11 GC strains with either all 11 isoforms of *opa* genes deleted (MS11ΔOpa) or the expression of a single nonphase variable Opa_52_ protein that binds to CEACAMs (MS11Opa_CEA_) at a MOI of 10 (10 bacteria per luminal epithelial cell) for 24 hours at 37°C. Since 10 of the 11 Opa isoforms in MS11 bind to CEACAMs (22, 23), Opa_52_ was chosen to represent CEACAM-binding Opas. Culture supernatants were collected to quantify cytokine secretion using ELISA and Luminex Magpix. Cytokine levels were normalized to the luminal surface area of tissue explants and the volume of supernatant. Uninoculated tissue explants secreted detectable or modest amounts of proinflammatory cytokines IL-1β (Figure 1A) and TNF-α (Figure 1B), the antiinflammatory cytokine IL-10 (Figure 1C), and the pleiotropic cytokine TGF-β (Figure 1D). Inoculation with MS11Opa_CEA_, which colonize the cervical epithelium more efficiently than MS11ΔOpa (16), significantly increased both the proinflammatory and antiinflammatory cytokines but not TGF-β (Figure 1, A–D, and Table 1). However, inoculation with MS11ΔOpa GC, which colonize less but penetrate more efficiently than MS11Opa_CEA_ (16), did not significantly elevate the level of these cytokines (Figure 1, A–D, and Table 1). IL-17A was undetectable in culture supernatants of GC uninoculated and inoculated cervical tissue explants (Figure 1E). When inoculating explants containing the ectocervix alone, the results were similar to those containing all 3 cervical regions (Figure 1, F–H, and Table 1). Inoculation with MS11ΔOpa GC slightly increased cytokines secreted by ectocervical tissue explants, like IL-1β and IL-10, but the increases were not statistically significant (Figure 1, F–H, and Table 1). Because the MS11Opa_CEA_ and ΔOpa variants reached similar numbers after 24-hour culture (Supplemental Figure 1; supplemental material available online with this article; https://doi.org/10.1172/JCI183331DS1), observed differences in cytokine responses cannot be due to growth differences. These data suggest that GC infection elevates the local secretion levels of both proinflammatory and antiinflammatory cytokines by the cervix during the first 24 hours of infection in an Opa_CEA_-dependent manner.

### GC-induced proinflammatory cytokine secretion in the human cervix depends on NF-κB.

**Figure 2:**
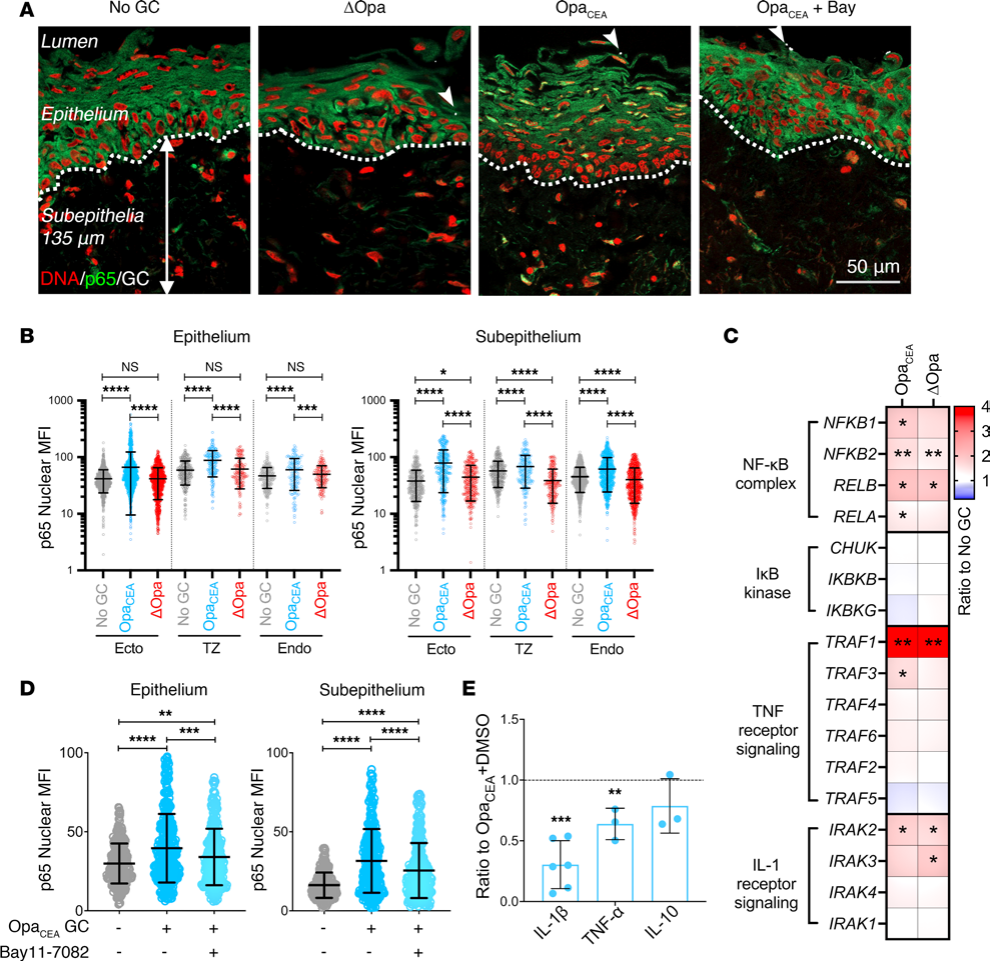
NF-κB is involved in GC-induced proinflammatory cytokine production by the human cervix. Human cervical tissue explants were incubated without or with MS11Opa_CEA_ or MS11ΔOpa (MOI ~ 10) in the absence or presence of the NF-κB inhibitor Bay11-7082 (3 μM) for 24 hours. (**A**) Representative CFM images of ectocervical tissue sections stained for NF-κB p65, GC, and nuclei. White dashed lines outline the epithelium, arrows indicate the 135μm depth of the subepithelium, and arrowheads point to GC attached to the epithelium. Scale bar: 50 μm. (**B**) The mean fluorescent intensity (MFI) (± SD) of NF-κB p65 in the nuclei of individual epithelial and subepithelial cells in the ectocervix (Ecto), transformation zone (TZ), and endocervix (Endo). Data were generated from cervical tissues from 3 to 4 individuals, 2 independent analyses per cervix, and 35–200 epithelial and 45–170 subepithelial cells per analysis per cervical region. (**C**) The heatmap shows relative mRNA levels of NF-κB–related genes by NanoString Immunology panel and compared MS11Opa_CEA_- or ΔOpa-inoculated with no GC control tissues from 3 individuals are shown. (**D**) Nuclear MFI of NF-κB p65 in individual epithelial and subepithelial cells from the ectocervical tissue explants inoculated without or with MS11Opa_CEA_ in the absence or presence of Bay11-7082. Data points represent individual nuclei. Average values (± SD) were generated from ectocervical tissues from 3 individuals, 3 independent analyses per cervix, and 40 nuclei per region per analysis. (**E**) Cytokine concentrations in the supernatants of ectocervical tissue explants by Luminex Magpix and ELISA. Shown are the ratios of cytokine concentrations (± SD) secreted by explants inoculated with MS11Opa_CEA_ in the presence of Bay11-7082 compared with the vehicle control DMSO. Data points represent individual analysis. *P < 0.05, **P < 0.01, ***P < 0.001, ****P < 0.0001, by 1-way ANOVA (**B**–**D**) and unpaired Student’s *t* test (**E**).

The transcription factor NF-κB is a crucial regulator of cytokine production (57, 58). To examine the possible involvement of NF-κB in GC-mediated cytokine expression, we determined if GC infection activated NF-κB in cervical cells using immunofluorescence microscopy. We measured the mean fluorescence intensity (MFI) of the NF-κB p65 subunit in the nuclei of individual cervical cells within the epithelium and subepithelium (135 μm below the epithelium) (Figure 2A and Supplemental Figure 2). MS11Opa_CEA_ increased NF-κB p65 MFI in the nuclei of both epithelial and subepithelial cells of all 3 cervical regions, ectocervix, transformation zone, and endocervix, compared with the no-GC controls (Figure 2B and Table 2). Among the 3 cervical regions of MS11Opa_CEA_-infected tissue explants, the increases in the nuclear NF-κB p65 MFI of the ectocervical epithelial and subepithelial cells appeared to be higher than the other two cervical regions (Figure 2B and Table 2). However, MS11ΔOpa did not significantly change the NF-κB p65 MFI of the cervical epithelial cell nuclei (Figure 2B and Table 2). Interestingly, MS11ΔOpa reduced the nuclear NF-κB p65 MFI of subepithelial cells in the two GC-penetrated cervical regions, while slightly increasing the nuclear NF-κB p65 MFI of ectocervical subepithelial cells where MS11ΔOpa cannot penetrate (Figure 2B and Table 2). Furthermore, both strains significantly increased the mRNA expression levels of the NF-κB genes *NFKB2* and *RELB* and genes encoding proteins that act upstream of NF-κB activation and downstream of TNF-α and IL-1β, *TRAF1*, and *IRAK2*, as measured by whole-tissue NanoString-based transcriptomic analysis (Figure 2C). MS11Opa_CEA_ also significantly elevated the mRNA levels of *NFKB1*, *RELA*, and *TRAF3* (Figure 2C). These data indicate that MS11Opa_CEA_ inoculation increases NF-κB activation levels of both cervical epithelial and subepithelial cells.

To determine if GC-induced NF-κB activation is involved in the cytokine induction, we utilized a membrane-permeable small-molecule inhibitor of NF-κB, Bay11-7082, that inhibits IκBα phosphorylation (59). The inhibitor (3 μM) was included in the 24-hour incubation of ectocervical tissue explants with MS11Opa_CEA_. Inhibitor treatment significantly reduced the NF-κB p65 MFI in the nuclei of ectocervical epithelial and subepithelial cells, even though the treatment did not bring their nuclear NF-κB p65 MFI back to the no-GC level (Figure 2D and Table 3). However, the NF-κB inhibitor did not significantly affect GC growth (Supplemental Figure 3). Notably, the inhibitor treatment significantly reduced the secretion levels of the inflammatory cytokines IL-1β and TNF-α in the ectocervix but did not have a significant effect on the ectocervical secretion of the antiinflammatory cytokine IL-10 (Figure 2E and Table 4). These data suggest that GC-induced proinflammatory but not antiinflammatory cytokine secretion involves NF-κB.

### Reductions in proinflammatory cytokines by NF-κB inhibition do not impact GC colonization of the ectocervix.

**Figure 3:**
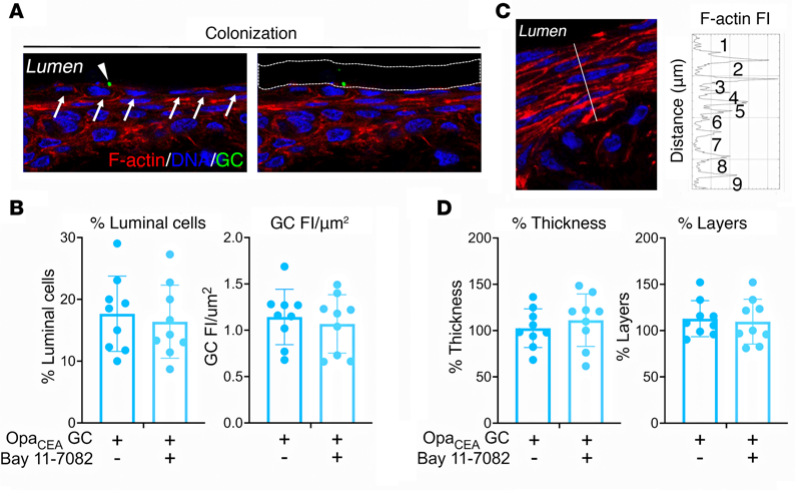
Proinflammatory cytokine reduction by NF-κB inhibition does not affect GC colonization of the ectocervix and ectocervical epithelial shedding. Human ectocervical tissue explants were inoculated without or with MS11Opa_CEA_ in the absence and presence of the NF-κB inhibitor Bay11-7082 (3 μM) for 24 hours (MOI ~ 10) and cryopreserved. Tissue sections were stained for GC by a polyclonal antibody, DNA by Hoechst, and F-actin by phalloidin and analyzed using CFM. (**A**) Quantification of GC colonization by the percentage of luminal epithelial cells associated with GC (left) and by fluorescence intensity (FI) of GC staining per μm^2^ of the luminal surface (right). Arrows point to individual luminal epithelial cells, and the arrowhead points to a GC-associated luminal epithelial cell. White dashed lines outline the luminal surface area where GC FI was measured. (**B**) The average percentages (± SD) of luminal epithelial cells associated with GC and the average value of GC FI per μm^2^ of the luminal surface from ectocervical tissues of 3 individuals with 3 independent analyses per cervix and 3–10 randomly acquired images per analysis. Data points represent each independent analysis. (**C**) Quantification of epithelium shedding by the percentage of remaining epithelium thickness (left, the length of the dashed line vertical against the epithelium) and cell layers based on the FI line profile of F-actin staining on the dashed line (right), compared with no GC control. (**D**) The average percentages (± SD) of the remaining epithelial thickness (left) and cell layers (right) from ectocervical tissues of 3 individuals with 3 independent analyses per cervix, 3–10 randomly acquired images per analysis, and 3 line profiles per image. Data points represent each independent analysis. There are no significant differences by unpaired Student’s *t* test.

Treatment with the proinflammatory cytokine IL-1β or TNF-α disrupts epithelial cell-cell junctions and induces epithelial cell apoptosis, in addition to activating inflammation (44, 47). We determined whether NF-κB inhibition, which reduced IL-1β and TNF-α production (Figure 2E), affected GC infection. Ectocervical tissue explants inoculated with MS11Opa_CEA_ in the absence or presence of the NF-κB inhibitor Bay11-7082 (3 μM) were stained for GC (antibodies), DNA (Hoechst), and F-actin (phalloidin) to mark each ectocervical cell (Figure 3A). We quantified GC colonization by measuring the percentage of luminal epithelial cells with attached GC (Figure 3A), reflecting the extent of the epithelium being infected, and GC fluorescence intensity (FI) per μm^2^ of the luminal surface (Figure 3A), reflecting the relative amount of GC colonizing the ectocervical epithelium. Treatment with the NF-κB inhibitor changed neither the percentage of luminal ectocervical epithelial cells associated with GC (Figure 3B) nor the GC FI per μm^2^ of the luminal surface significantly (Figure 3B). The NF-κB inhibitor also did not significantly affect GC growth (Supplemental Figure 3). We further quantified ectocervical epithelial cell shedding by measuring the percentage of the epithelial thickness and the remaining epithelial cell layers compared to no-GC control (100%) (Figure 3C). NF-κB inhibitor treatment did not affect the thickness (Figure 3D) or the cell layer number (Figure 3D) of the ectocervical epithelium. Even though the secreting levels of IL-1β and TNF-α were elevated in the absence of the NF-κB inhibitor (Figure 1, A and B), MS11Opa_CEA_ GC inoculation did not induce significant changes in epithelial thickness and cell layers (Figure 3D). Together, these data suggest that GC-induced proinflammatory cytokines do not interfere with GC colonization of the human cervix.

### The antiinflammatory cytokine IL-10 promotes GC colonization by inhibiting epithelial cell shedding.

**Figure 4:**
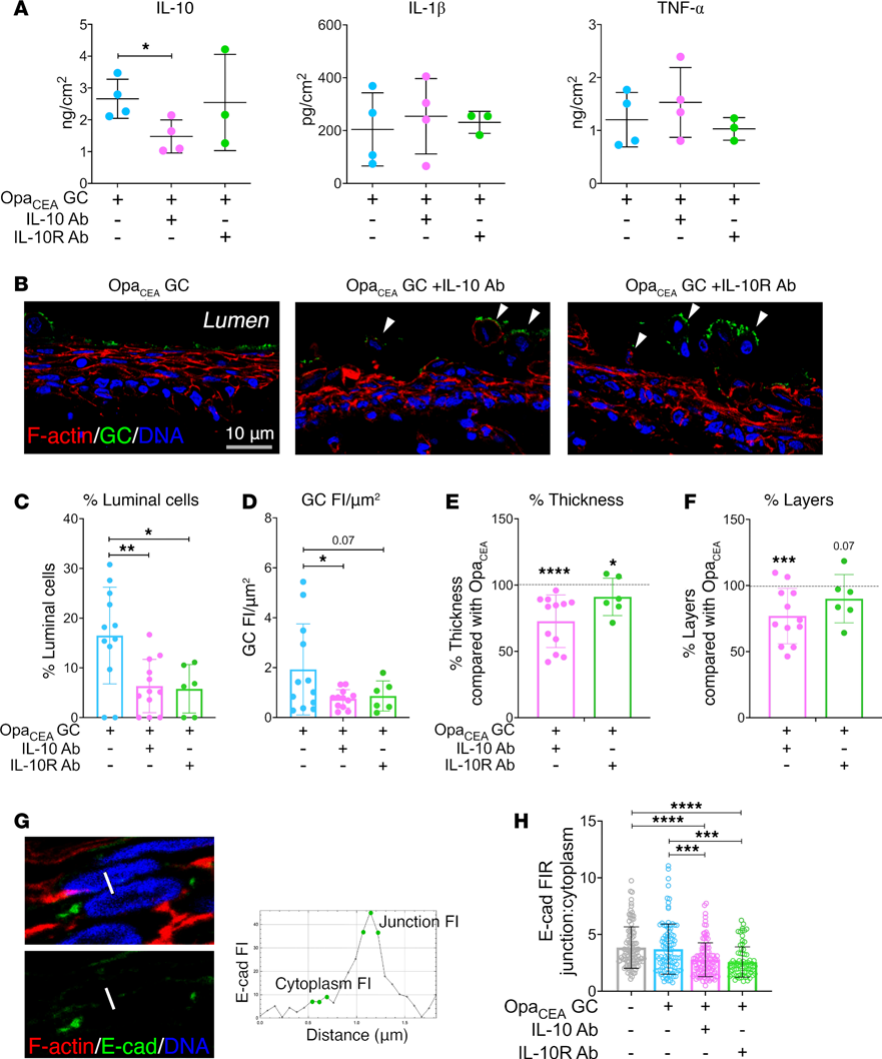
IL-10 neutralization and IL-10 receptor–blocking antibodies reduce GC colonization by increasing ectocervical epithelial cell shedding. Human ectocervical tissue explants were incubated without and with MS11Opa_CEA_ in the absence and presence of anti–IL-10 (10 μg/mL) or IL-10Rα antibody (5 μg/mL) for 24 hours (MOI ~ 10). The culture supernatants were collected, and tissues were cryopreserved. (**A**) The concentrations of IL-10, IL-1β, and TNF-α in the supernatants were measured by Luminex Magpix. Data points represent individual cervixes. *n* = 3–4. (**B**) Representative images of ectocervical tissue sections stained for GC, DNA, and F-actin. Arrowheads point to shed epithelial cells. Scale bar: 10 μm. (**C** and **D**) Quantification of GC colonization by the percentage (± SD) of luminal epithelial cells associated with GC (**C**) and by FI (± SD) of GC staining per μm^2^ of the luminal surface (**D**). Shown are the average values from 2 to 4 cervixes, 3 independent analyses per cervixes, and 4–8 randomly acquired images per analysis. Data points represent independent analysis. (**E** and **F**) Quantification of epithelium shedding by the percentages (± SD) of remaining epithelium thickness (**E**) and cell layers (**F**) in tissue explants inoculated with MS11Opa_CEA_ in the presence of IL-10 or IL-10Rα antibody, compared with tissue explants inoculated with MS11Opa_CEA_ without IL-10 or IL-10Rα antibody. Shown are the average values from 2 to 4 cervixes and 3 independent analyses per cervix. Data points represent independent analysis. (**G** and **H**) Disruption of epithelial cell-cell junctions was determined by the FI ratio (FIR) of E-cadherin (E-cad) at the cell-cell border relative to the cytoplasm (**H**) using the average FI of 3 data points (green dots) along line profiles (**G**). Shown are the average FIR (± SD) from 2 to 4 cervixes with 3 independent analyses per cervix, 4–10 randomly acquired images per analysis, and 3 line profiles per image. Data points represent individual line profiles. *P < 0.05, **P < 0.01, ***P < 0.001, ****P < 0.001, by 1-way ANOVA.

In contrast to the proinflammatory cytokines IL-1β and TNF-α, the antiinflammatory cytokine IL-10 has been shown to strengthen the barrier and wound repair functions of the epithelium (44, 51, 52). We determined whether MS11Opa_CEA_-induced IL-10 production contributes to GC infection, using either an IL-10 or IL-10Rα antibody to prevent secreted IL-10 from engaging IL-10R (60, 61). Ectocervical tissue explants were inoculated with MS11Opa_CEA_ in the absence or presence of IL-10 (10 μg/mL) or IL-10Rα (5 μg/mL) antibodies for 24 hours. Luminex Magpix and ELISA analysis found that the IL-10 antibody effectively reduced the IL-10 level but did not significantly affect the levels of the proinflammatory cytokines IL-1β and TNF-α in the supernatants of ectocervical tissue explants inoculated with MS11Opa_CEA_ (Figure 4A). IL-10 and IL-10Rα antibodies did not directly affect GC viability (Supplemental Figure 4). Immunofluorescence microscopic analysis found that the IL-10Rα antibody concentrated primarily at the ectocervical epithelium but could also be detected in the subepithelium (Supplemental Figure 5). Importantly, both IL-10 and IL-10Rα antibodies significantly reduced the percentages of the luminal epithelial cells associated with GC (Figure 4, B and C) and the GC FI per μm^2^ (Figure 4, B and D), indicating reductions in GC colonization. Increased disassociation of GC-attached epithelial cells from the ectocervix was clearly visible in immunofluorescence images of IL-10 or IL-10Rα antibody-treated tissue explants, compared with untreated tissue explants (Figure 4B and Supplemental Figure 6, arrowheads). We quantified epithelial cell shedding using confocal fluorescence microscope (CFM) images. Treatment with IL-10 and IL-10Rα antibodies resulted in reduced epithelial thickness (Figure 4E) and cell layer number (Figure 4F), as compared with untreated MS11Opa_CEA_-infected ectocervical tissue explants as controls, indicating increased epithelial cell shedding. Using CFM images, we explored if IL-10 neutralization or IL-10Rα blocking induced epithelial shedding by disrupting epithelial cell-cell junction. We measured the FI ratios of the ectocervical epithelial cell-cell junctional protein E-cadherin at the cell-cell junction relative to the cytoplasm using FI line profiles that vertically cross the epithelial cell-cell contact surface (Figure 4G). Treatment with IL-10 or IL-10Rα antibodies significantly reduced the junction to cytoplasm ratios of E-cadherin FI (Figure 4H). Our results suggest that MS11Opa_CEA_-induced IL-10 promotes bacterial colonization by strengthening epithelial cell-cell junctions and preventing epithelial cell shedding.

### GC induce IL-10 production by engaging CEACAM1 and activating SHP1/2.

**Figure 5:**
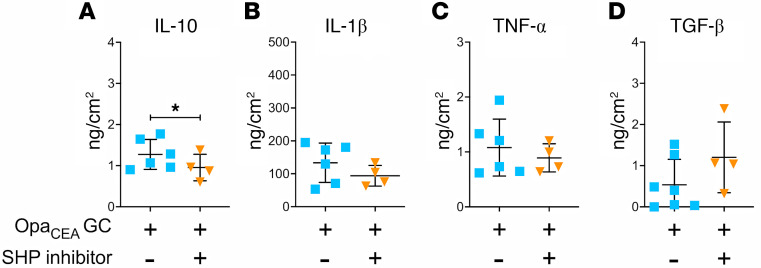
GC-induced IL-10 but not IL-1β and TNF-α secretion by human cervical tissue explants requires CEACAM1 signaling. Supernatants of cervical tissue explants inoculated with MS11Opa_CEA_ in the absence or presence of the SHP1/2 inhibitor NSC-87877 (20 μM) for 24 hours were collected. The cytokine concentrations of IL-10 (**A**), IL-1β (**B**), TNF-α (**C**), and TGF-β (**D**) were measured by Luminex Magpix or ELISA (TGF-β) and normalized to the luminal surface area and the supernatant volume of each explant. Shown are the average concentration (± SD) from 4 to 7 cervixes. Data points represent individual cervixes. *P < 0.05, by unpaired Student’s *t* test.

Our previous studies have shown that GC interaction with CEACAM1 via Opa_CEA_ increases GC colonization at the ectocervix by inhibiting epithelial cell shedding. This increase depends on the ITIM motif within the cytoplasmic domain of CEACAM1 and subsequent activation of SHP1/2 (16). Since MS11Opa_CEA_ but not MS11ΔOpa increase cervical secretion of IL-10, we questioned whether GC induce IL-10 production through CEACAM1. We treated ectocervical tissue explants with NSC-87877, a membrane-permeable small-molecule inhibitor of SHP1/2 (62) that inhibits the effects of Opa_CEA_ on GC colonization and cervical epithelial shedding (16). We collected the supernatants from explants inoculated without or with MS11Opa_CEA_ in the absence or presence of NSC-87877 (20 μM) and determined the concentration of IL-10, IL-1β, TNF-α, and TGF-β using Luminex Magpix or ELISA. Treatment with the SHP1/2 inhibitor significantly reduced the secreted IL-10 level by MS11Opa_CEA_-inoculated tissue explants compared with tissue explants without the inhibitor (Figure 5A and Table 5). However, treatment of the SHP1/2 inhibitor did not significantly affect the secretion levels of the proinflammatory cytokines IL-1β and TNF-α and the pleiotropic cytokine TGF-β (Figure 5, B–D, and Table 5). This result suggests that GC induce IL-10 production but not IL-1β and TNF-α through CEACAM1 and the downstream SHP1/2, and GC-induced IL-10 contributes to Opa_CEA_-mediated colonization enhancement.

## Discussion

This study utilized human cervical tissue explants as an infection model to demonstrate that GC inoculation increases the secretion levels of both the proinflammatory cytokines IL-1β and TNF-α and the antiinflammatory cytokines IL-10 but not TGF-β and IL-17A during the first 24 hours of infection. The locally secreted antiinflammatory cytokine IL-10, but not inflammatory cytokines IL-1β and TNF-α, modulates GC infection at the human cervix during this period. GC increase the secretion of IL-1β and TNF-α, but not IL-10, by activating NF-κB in both cervical epithelial and subepithelial cells. GC elevate the secretion of IL-10 but not IL-1β and TNF-α by binding to CEACAMs and activating their downstream SHP1/2. GC-induced IL-10 enhances GC colonization by inhibiting epithelial cell-cell junction disassembly and GC-associated cervical epithelial cell shedding.

A major finding of this study is that during the first 24 hours of infection, GC colonization elevates the local secretion of the antiinflammatory cytokine IL-10 at the cervix. While IL-10 was previously detected in women’s cervical mucus, its level was reported to be increased, decreased, or unchanged by GC infection (31, 33, 34). However, in these studies, how long women had been infected before sample collection and whether infected women were symptomatic or asymptomatic were unknown. GC has been shown to elevate IL-10 levels when cultured with human monocyte-derived dendritic cells (35) and macrophages (37), CD4^+^ T cells (38), and mononuclear cells (39) from human peripheral blood. However, during the initial stage of natural infection (<24 hours after exposure), GC primarily colonize the ectocervical epithelium (16) and have little chance of encountering subepithelial immune cells. Since the human cervical tissue explants are surgically disconnected from the blood and lymphatic circulation, our data provide the first evidence to our knowledge for GC-elevated mucosal secretion of IL-10 by human cervical cells. These data together demonstrate the robust ability of GC to induce the production of IL-10, a potent antiinflammatory cytokine, by the local mucosal or central immunity, depending on the types of cells they encounter. Whether GC-enhanced local secretion of IL-10 is sufficient to suppress inflammation activation associated with symptomatic infection is unknown. A major limitation of the current cervical tissue explant model is that it does not allow us to determine if the local cytokine response induces or suppresses the infiltration of neutrophils from the blood circulation. However, based on the potent inflammation inhibitory function of IL-10 (27), GC-induced elevation of IL-10 secretion at the cervix during the first 24 hours of infection would negatively regulate local inflammation activation, consequently reducing blood immune cell infiltration and facilitating GC colonization.

This study found that cervical tissue explants constitutively secreted IL-10 at levels relatively higher than the basal level of the inflammatory cytokines IL-1β and TNF-α. Even though tissue wounding caused by surgery, tissue handling, and initial antibiotic treatment could modulate cytokine production, the relatively high basal level of IL-10 suggests an antiinflammatory cytokine environment of the human cervix. This environment is consistent with the physiological functions of the cervix, a gate between the vagina and the uterus, which requires the cervix to tolerate semen and microbiota. The FRT is known to upregulate IL-10 production during pregnancy, which is critical for the immune tolerance of fetuses (63, 64). Here, we found that GC can further enhance the local production of IL-10 in the cervix. Furthermore, IL-10 neutralization and receptor blocking drastically increased epithelial shedding, but reductions in inflammatory cytokines did not. These results further support the notion that the antiinflammatory cytokine environment of the cervix, particularly the locally secreted IL-10, may effectively suppress the epithelial disrupting function of IL-1β and TNF-α, even when GC elevate the levels of IL-1β and TNF-α. However, this hypothesis requires further investigation.

Our results suggest that locally secreted IL-10 can directly act on cervical epithelial cells to promote GC colonization by inhibiting the disassembly of epithelial cell-cell junctions and GC-associated epithelial cell shedding. There are several potential mechanisms by which IL-10 can promote GC colonization at the cervix: activating epithelial cell proliferation, differentiation programs, and wound repair pathways by directly engaging IL-10R on epithelial cells (41, 43); inhibiting proinflammatory cytokine production; and interfering with inflammatory cytokine-induced signaling pathways (27, 41) and mediated functions, including epithelial junction disrupting function (45, 49, 65). Here, we show that IL-10 neutralization or IL-10Rα blocking by antibodies weakens the epithelial cell-cell junction but does not increase the secretion of IL-1β and TNF-α by the cervix, suggesting that the observed effect of IL-10 is not mediated through downregulating the secretion of inflammatory cytokines but via interfering with the epithelial junction-disrupting function of imflammatory cytokines. However, locally secreted IL-10 may inhibit inflammatory cytokine production in vivo. The unchanged levels of inflammatory cytokines we observed could be due to the failure of IL-10 and IL-10Rα antibodies to reach inflammatory cytokine-producing cells in the subepithelium of the cervix. The primary location of IL-10Rα blocking antibody at the luminal layer of the ectocervical epithelium suggests that IL-10Rα antibody only blocked IL-10 from binding IL-10R at the luminal surface of cervical epithelial cells. IL-10–mediated inhibition of shedding GC-associated cervical epithelial cells in the presence of the potent inflammatory cytokines IL-1β and TNF-α underscores the importance of the direct interaction of IL-10 with IL-10R on epithelial cells for GC-establishing infection at the human cervix.

The elevated levels of inflammatory cytokines and nuclear NF-κB, a crucial transcriptional factor that controls the production of inflammatory cytokines (57, 58), by GC inoculation suggest that GC cannot escape immune detection. Instead, GC increase the secretion of antiinflammatory cytokines to counteract inflammation activation and maintain or even enhance immune tolerance in the cervix, depending on the relative levels of proinflammatory and antiinflammatory cytokines. GC can activate cervical cell signaling, such as TLR and inflammasome pathways (66, 67), through surface molecules, including pili, LOS, and peptidoglycans (68–72), leading to NF-κB activation. Interestingly, MS11Opa_CEA_, which primarily colonize the luminal surface of the cervical epithelial cells, activate NF-κB in both epithelial and subepithelial cells, suggesting the activation of NF-κB in subepithelial cells may be indirect, probably through inflammatory cytokines secreted by cervical epithelial cells. Surprisingly, MS11ΔOpa, which can penetrate into the subepithelium of the transformation zone and the endocervix and may directly interact with subepithelial immune cells, do not significantly elevate IL-1β, TNF-α, and IL-10 at the first 24-hour window. The weak local cytokine response induced by MS11ΔOpa is associated with a lower level of increase in NF-κB in the nuclei of epithelial cells and even reductions in NF-κB in the nuclei of subepithelial cells. As MS11Opa_CEA_ was generated by introducing an Opa_CEA_ to MS11ΔOpa (73) and showed similar growth rates, our data suggest a requirement of the Opa-CEACAM interaction to induce IL-1β and TNF-α production. However, inhibition of the CEACAM1 downstream molecule SHP1/2 slightly but not significantly reduced MS11Opa_CEA_-induced IL-1β and TNF-α, which argues against this notion. Besides activating SHP1/2, Opa-CEACAM interactions enhance GC colonization efficiency at the cervix (16), increasing the number of GC that interact with epithelial cells directly, which potentially enables higher levels of TLR, inflammasome, and cervical cell signaling activation, leading to their downstream NF-κB activation and proinflammatory cytokine secretion. This multifaceted hypothesis remains to be tested. On the other hand, MS11ΔOpa-induced NF-κB reduction in the nuclei of the subepithelial cells suggests an Opa-independent mechanism for GC suppression of inflammatory responses.

This study suggests that GC elevate the IL-10–secreting levels in the human cervix by engaging CEACAM1, which contains an ITIM motif in the cytoplasmic tail and can activate SHP1/2 downstream. Our supporting data include the induction of IL-10 by MS11Opa_CEA_ but not MS11ΔOpa and reductions of both IL-10 secretion and MS11Opa_CEA_ cervical colonization (16) by the SHP1/2 inhibitor. Which types of cervical cells respond to CEACAM1 binding to secret IL-10 remains unknown. Immune cells are the primary sources of IL-10, but nonimmune cells, such as epithelial cells, can also secrete IL-10 (43, 74). In addition to cervical epithelial cells (16), many types of immune cells express CEACAM1 (75), including T cells, where it functions as a negative regulator (76–78). CEACAM1 knockout in mice leads to the hyperexpansion of conventional T cells but the reduction of regulatory T cells, IL-10 producers, in the liver (79). In vitro experiments have shown that GC inhibits T cell receptor–mediated human T cell activation by engaging CEACAM1 on the T cell surface by Opa and activating CEACAM1 downstream molecule SHP1/2, downregulating T cell receptor signaling (80, 81). GC has also been shown in vitro to promote macrophage polarization toward an alternatively activated phenotype characterized by heightened IL-10 production (37). Accumulating evidence supports Opa-CEACAM1 interactions as one of the mechanisms for GC to induce IL-10 production. However, this does exclude other possible mechanisms for GC to regulate IL-10 production in the cervix.

Many human factors can influence the cytokine production of the cervix, such as donor ethnic background, age, microbiome, hormonal cycle, and clinical history, which are likely the sources of our data variability. Microbiomes and female sex hormones have all been shown to regulate the mucosal immunity of the FRT (82, 83). This study is just the beginning of the exploration of the relationship between the local cytokine responses and GC infection. Our human cervical explant model and the results of this study provide a step forward to our future study of how human cervical microbiome and female sex hormones regulate the local cytokine response to GC infection.

Our study reveals a mechanism GC employ to evade mucosal immunity for colonization, enhancing local secretion of the antiinflammatory cytokine IL-10 at the mucosal surface of the cervix by engaging CEACAM1. IL-10 then directly acts on cervical epithelial cells, preventing GC-colonizing epithelial cells from shedding off, in addition to its immune-suppressing function. Our results lead to a hypothesis that GC induce early and local IL-10 production in the cervix to promote asymptomatic infection in women. This hypothesis potentially explains why CEACAM-binding Opa proteins have been evolutionally favored (22, 23).

## Methods

### Sex as a biological variable.

Our study exclusively examined *N*. *gonorrhoeae* infection of the human cervix. Cervical tissues from female donors were used. Human cervical tissues used were anonymized. No race or ethnic information was provided.

### Neisseria strains.

*N*. *gonorrhoeae* strain MS11 with all 11 *opa* gene deleted (ΔOpa) and MS11 ΔOpa expressing nonphase variable CEACAM-binding Opa_52_ (Opa_CEA_) (73) were used. Piliated bacteria were identified based on colony morphology using a dissecting light microscope. GC were grown on plates with GC media and 1% Kellogg’s supplement (GCK) for 16–18 hours before inoculation. The concentration of bacteria in suspension was determined by using a spectrophotometer.

### Bacterial growth rates.

MS11Opa_CEA_ and ΔOpa were cultured in CMRL-1066 media containing 5% FBS for 0, 6, 12, and 24 hours in the absence and presence of the NF-κB inhibitor Bay11-7082, DMSO, IL-10 neutralization antibodies (10 μg/mL), or IL-10Rα–blocking antibodies (5 μg/mL). Bacteria were enumerated by counting CFU after serially diluting tissue culture media and plating on GCK plates or quantified by optical density at 650 nm.

### Human cervical tissue explants.

Healthy cervical tissues were obtained from donors (28–42 years old) undergoing hysterectomies for medical reasons unrelated to the cervix and received within 24 hours after surgery through the National Disease Research Interchange. In addition to the donors’ age, a pathology report of the cervix came with each tissue shipment. Only cervical tissues without pathological changes were used. Tissue explants were generated and processed using a previously published protocol (84). Briefly, muscle parts of the tissue were removed using a carbon steel surgical blade. Cervical tissues from 1 individual were cut into 3 or 4 equal pieces with the dimension of approximately 2.5 cm (L) × 0.6 cm (W) × 0.3 cm (H) for comparing different inoculation conditions. For ectocervix tissue explants, ectocervical tissues were separated from the transformational zone and endocervix by visual inspection. Tissue explants were incubated in the CMRL-1066 (11530037, Gibco), containing 5% heat-inactivated fetal bovine serum (A5256701, Gibco), L-glutamine (2 mM, 25030081, Gibco), bovine insulin (1 μg/mL, 16634, Sigma-Aldrich), and penicillin/streptomycin for 24 hours, followed by antibiotic-free media for another 24 hours. GC were inoculated at a MOI approximately 10 (10 bacteria to 1 luminal epithelial cell). The number of epithelial cells at the luminal surface was determined by the luminal surface area of individual explants divided by the average luminal area of individual cervical epithelial cells (25 μm^2^). The cervical tissue explants were incubated with GC at 37°C with 5% CO_2_ with gentle shaking for 24 hours and washed with antibiotic-free cervical tissue culture medium at 6 and 12 hours to remove nonadhered bacteria.

### Inhibitor and antibody treatment.

Cervical tissue explants were incubated with bacteria in the presence or absence of NF-κB inhibitor BAY 11-7082 (3 μM, HY-13453, MedChemExpress) (59), SHP inhibitor NSC-87877 (85) (20 μM, 565851-50MG, EMD Millipore), rat anti-human IL-10 antibody (10 μg/mL, 10100-01, SouthernBiotech), or rat anti-human IL-10Rα antibody (5 μg/mL, 308802, BioLegend) for 24 hours. The inhibitors and the antibodies were replenished after the 6 and 12 hours washing.

### Quantification of cytokine secretion.

Supernatants collected from cervical tissue explants at 6, 12, and 24 hours after inoculation were pooled together in an 1:1:2 volume ratio, and a protease inhibitor cocktail (PIC0002, Sigma-Aldrich) was added to prevent protein degradation. Supernatants collected from cervical tissue explants used for previous studies on GC infectivity and infection mechanism (16) were utilized for cytokine quantification for Figure 1, A–E, and Figure 5. Cytokines in the supernatants were quantified using a Luminex Magpix system (Bio-Plex MAGPIX Multiplex Reader, Bio-Rad Laboratories) and the Human XL Cytokine Luminex Performance Assay 46-Plex Fixed Panel (Bio-Techne), according to the manufacturer’s protocol. The secretion levels of IL-1β and TGF-β were quantified using ELISA with the human IL-1β ELISA Max Deluxe kit (437004, BioLegend) and human TGF-β1 duoset ELISA kit (Dy204-05, R&D System). Each sample was run in duplicate. The cytokine concentrations of each sample by either Luminex Magpix or ELISA were normalized based on the supernatant volume and the tissue luminal surface area.

### Immunofluorescence analysis of human cervical tissue explants.

The tissue explants were fixed in 4% paraformaldehyde 24 hours after inoculation; embedded in 20% gelatin; cryopreserved; sectioned crossing the luminal, basal surfaces of the epithelium and subepithelial tissues; stained for F-actin (100 nM, phalloidin, PHDH1, Cytoskeleton), E-cadherin (mouse anti-human E-cadherin antibody, 5 μg/mL, 610182, BD Bioscience), NF-κB p65 (rabbit anti-human p65 antibody, 5 μg/mL, 8242, Cell Signaling Technology), and GC (custom polyclonal antibody) (84) by specific antibodies and nuclei by Hoechst (20 μg/mL, H3570, Life Technologies); and imaged using CFM (Zeiss LSM 980, Carl Zeiss Microscopy LLC). Images were acquired using Zeiss Zen software.

The levels of NF-κB activation were quantified by the MFI of p65 staining in individual nuclei. Individual nuclei were identified by Hoechst staining using NIH ImageJ. The data were generated using 3–4 human cervixes, 2 independent analyses per cervix, 5–11 randomly acquired images per ectocervical region, 3–8 randomly acquired images per transformation zone region, 3–10 randomly acquired images per endocervical region per analysis, including 200 nuclei per ectocervical epithelial region, 70 nuclei per ectocervical subepithelial region, 35 nuclei per transformation zone epithelial region, 45 nuclei per transformation zone subepithelial region, 35 nuclei per endocervical epithelial region, and 170 nuclei per endocervical subepithelial region per analysis.

The levels of GC colonization at the ectocervix were quantified by two methods using CFM images (16): (a) the percentage of luminal epithelial cells with GC attached at the luminal surface by manually accounting and (b) the FI of GC staining per μm^2^ of the luminal surface using the NIH ImageJ software. The data were generated from 2–4 human cervixes, 3 independent analyses per cervix, and 3–10 randomly acquired images per analysis.

The levels of epithelial shedding in the ectocervix were determined by two methods (16): (a) the percentage of the remaining thickness (μm) of the epithelium and (b) the percentage of remaining epithelial cell layers (based on F-actin staining) compared with uninfected ectocervical tissue explants, using the NIH ImageJ software. The data were generated from 2–4 human cervixes, 3 independent analyses per cervix, and 3–10 randomly acquired images per analysis.

The redistribution of E-cadherin from the cell-cell junction to the cytoplasm was evaluated by the FI ratios of E-cadherin staining at the cell-cell junction relative to the cytoplasm in individual epithelial cells using CFM images by the NIH ImageJ software. The data were generated from 2–4 human cervixes, 3 independent analyses per cervix, and 4–10 randomly acquired images per analysis.

### Gene expression analysis by NanoString.

At 24 hours after inoculation, the tissue explants were embedded in Tissue-Tek O.C.T. Compound (4583, Sakura Finetek) and snap froze in liquid nitrogen. Thirty 10-μm thickness tissue sections were collected from each tissue explant for RNA isolation using the RNeasy Mini Kit (74104, Qiagen). The multiplexed NanoString nCounter Immunology Panel (PSTD Hs Immunology V2-12, NanoString Technologies) with 594 genes was used according to the manufacturer’s protocol. The data quality control, background threshold, normalization, and differential gene expression analysis were conducted using nSolver Analysis software. The quality of the run for each sample was confirmed by the quality control, including the 6 spiked-in RNA-positive controls and the 8 negative controls present in the panel, the fields of view per sample counted, and the binding density. Gene expression data were normalized by using the 15 housekeeping genes present in the panels. Background level was determined by mean counts of 8 negative control probes plus 2 SDs. When a gene’s raw counts were below the background of >50% of all samples, this gene was excluded from differential expression analysis. Differential gene expression analysis was performed by comparing the normalized count of each gene in infected samples and uninfected samples, and genes with *P* < 0.05 were identified as differential expression genes.

### Statistics.

Statistical significance was assessed using the 2-tailed Student’s *t* test and 1-way ANOVA by Prism software (GraphPad). A *P* value of less than 0.05 was considered significant.

### Study approval.

Human cervical tissues were obtained through the National Disease Research Interchange (Philadelphia, Pennsylvania, USA). Human cervical tissues used were anonymized. No race or ethnic information was provided. The use of human tissues has been approved by the Institution Review Board of the University of Maryland.

### Data availability.

Values for all data points in graphs are reported in the Supporting Data Values file.

## Author contributions

WS conceptualized the study. YD, VLE, and QY performed experiments and analyzed data. HT analyzed transcriptomic data. DCS generated genetically modified bacterial strains. WS and YD wrote the manuscript. DCS, HT, and VLE reviewed and edited the manuscript. All authors approved the final version of the manuscript.

## Supplementary Material

Supplemental data

Supporting data values

## Figures and Tables

**Table 4:**
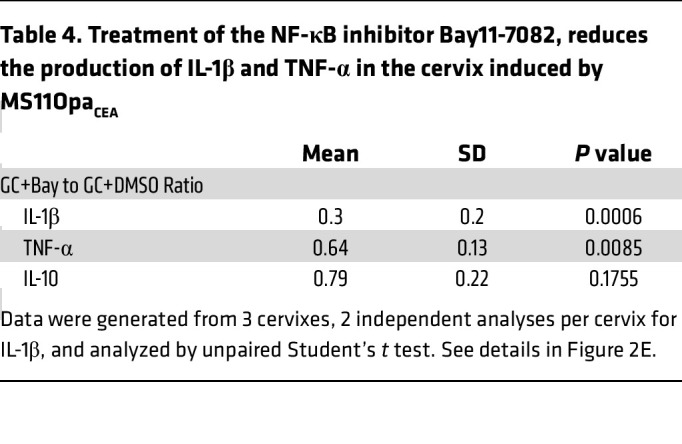
Treatment of the NF-κB inhibitor Bay11-7082, reduces the production of IL-1β and TNF-α in the cervix induced by MS11Opa_CEA_

**Table 2:**
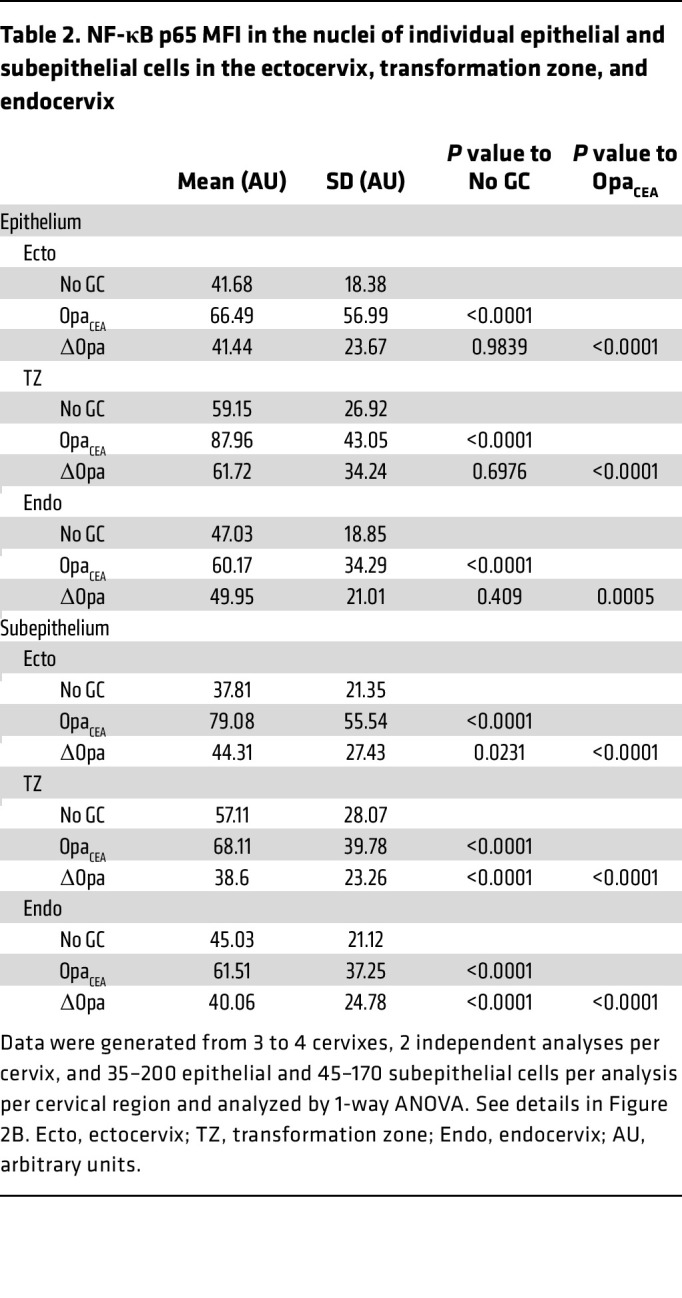
NF-κB p65 MFI in the nuclei of individual epithelial and subepithelial cells in the ectocervix, transformation zone, and endocervix

**Table 1:**
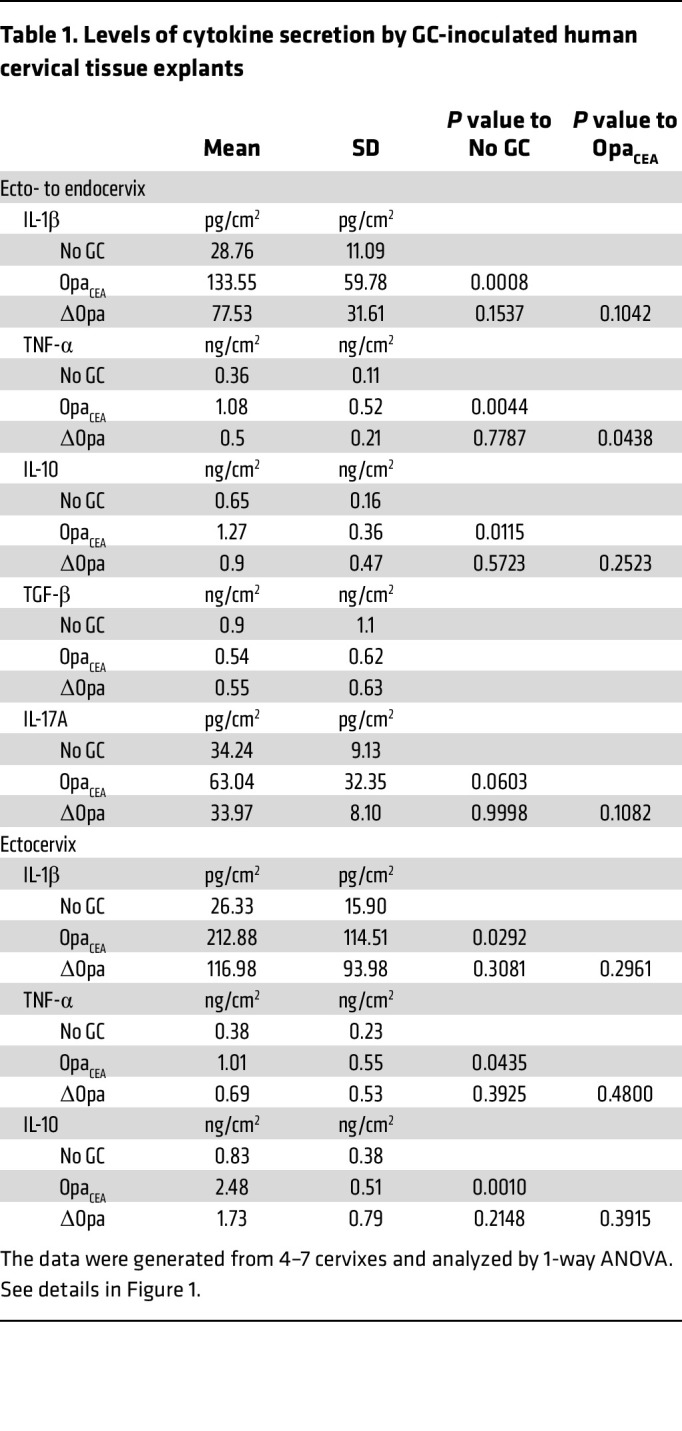
Levels of cytokine secretion by GC-inoculated human cervical tissue explants

**Table 3:**
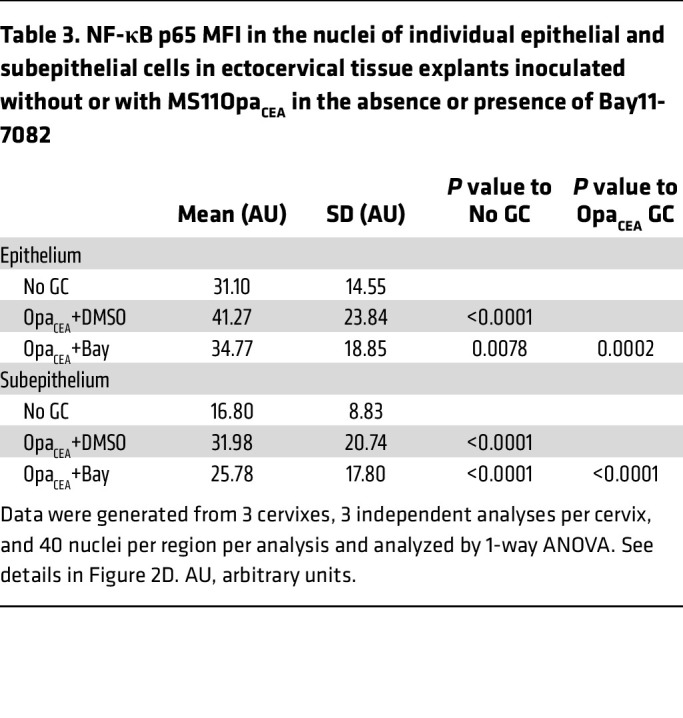
NF-κB p65 MFI in the nuclei of individual epithelial and subepithelial cells in ectocervical tissue explants inoculated without or with MS11Opa_CEA_ in the absence or presence of Bay11-7082

**Table 5:**
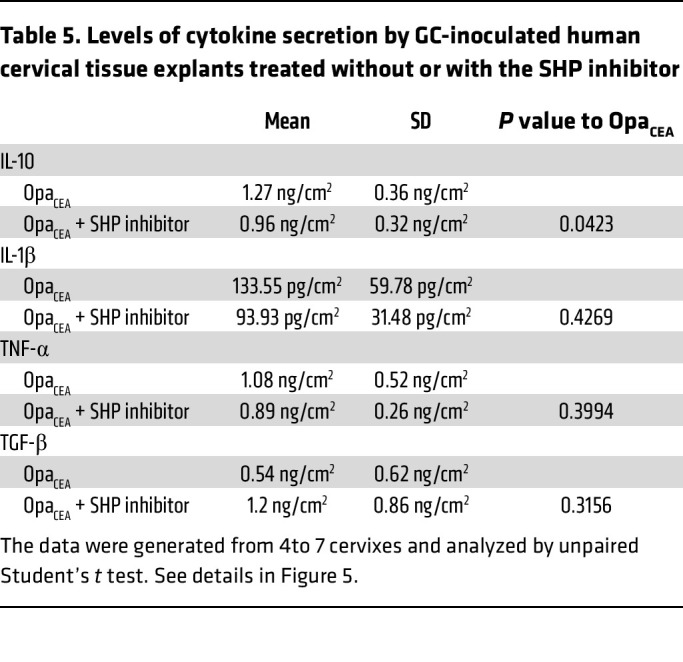
Levels of cytokine secretion by GC-inoculated human cervical tissue explants treated without or with the SHP inhibitor

## Version 1 11/25/2024

In-Press Preview

## Version 2 01/16/2025

Electronic publication
