# Supplementary material for: Neisseria gonorrhoeae induces local secretion of IL-10 at the human cervix to promote colonization
Source: J Clin Invest. 2024 Nov 14;135(2):e183331. doi: 10.1172/JCI183331 (PMC11735093; doi:10.1172/JCI183331)
Supplement: Supplemental data [file jci-135-183331-s050.pdf]

Supplementary Materials

6 Supplementary Figures

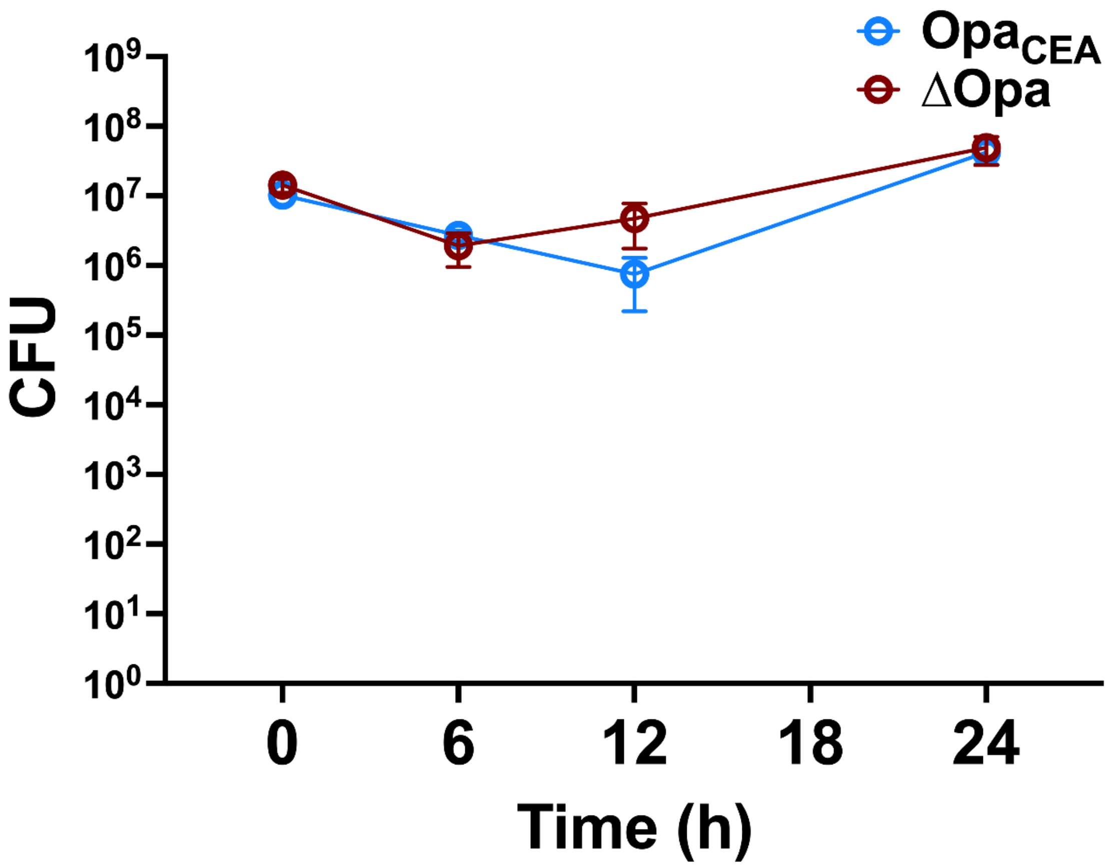

Supplementary Figure 1. MS11Opa_{CEA} and ΔOpa reached similar numbers after being cultured for 24 h. MS11Opa_{CEA} and ΔOpa were cultured in CMRL-1066 media containing 5% FBS for 0, 6, 12, and 24 h. Bacteria were enumerated by counting CFU after serially diluting tissue culture media and plating on GCK plates. Shown are the averages (±SD) of 3 independent experiments. There are no statistically significant differences by pair student's *t*-test.

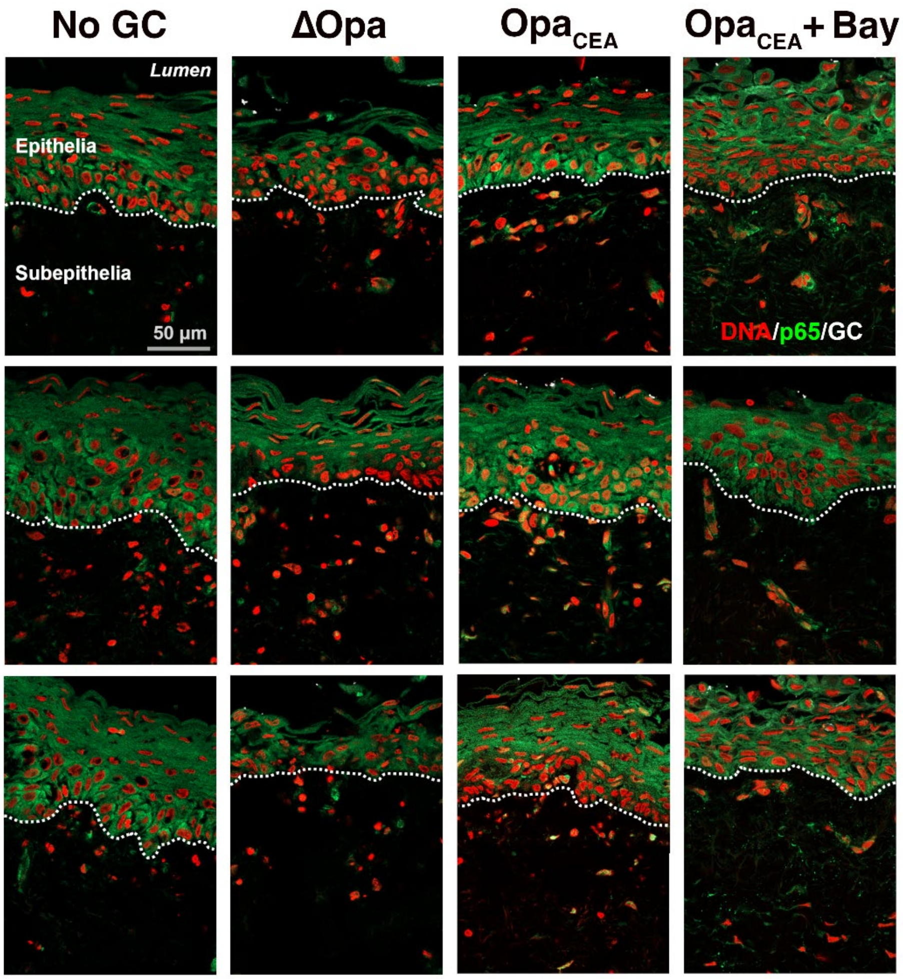

Supplementary Figure 2. MS11Opa$_{CEA}$ increase the nuclear level of NF-κB p65 in the ectocervix, and treatment of an NF-κB inhibitor reduces the increase. Human cervical tissue explants were incubated without or with MS11Opa$_{CEA}$ or MS11ΔOpa (MOI~10) in the absence or presence of the NF-κB inhibitor Bay11-7082 (3 μM) for 24 h. The tissues were cryopreserved. Tissue sections were stained for NF-κB p65 and GC by antibodies and nuclei by Hoechst and imaged using a confocal fluorescence microscope (CFM). Three sets of example images from ectocervical tissues without or with MS11ΔOpa or Opa$_{CEA}$ GC inoculation and the NF-κB inhibitor Bay11-7082 are shown. White dished lines outline the epithelium. Scale bar, 50 μm.

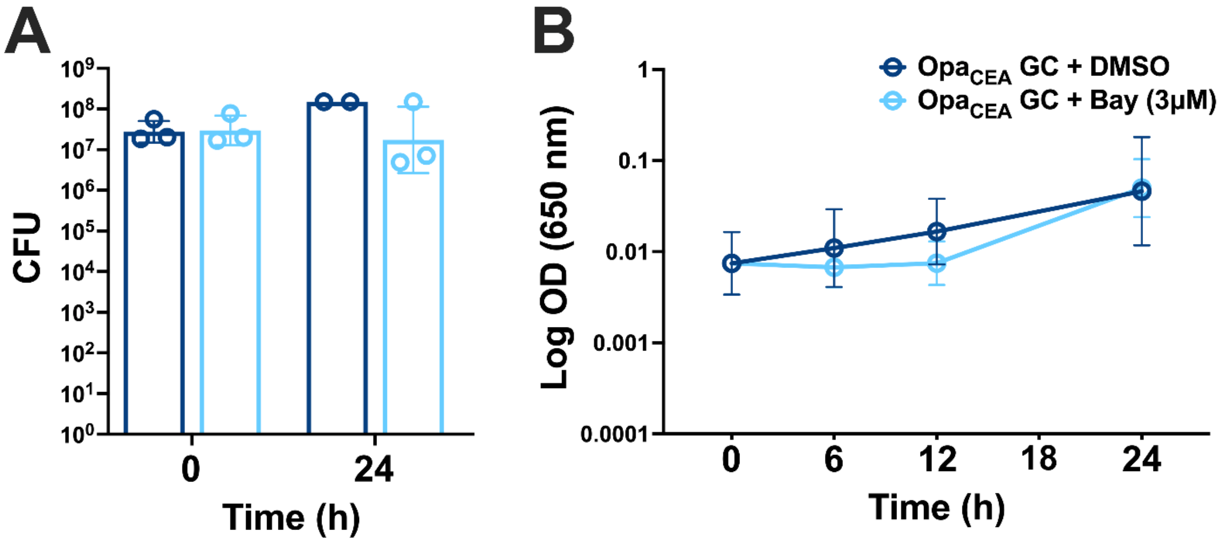

Supplementary Figure 3. Treatment of the NF-κB inhibitor Bay 11-7082 has no significant effect on GC growth. (**A**) Human ectocervical tissue explants were inoculated with MS11Opa$_{CEA}$ (MOI~10) in the absence and presence of the NF-κB inhibitor Bay11-7082 (3 μM) for 24 h with washing at 6 and 12 h. Bacteria in culture media at 24 h were enumerated by counting CFU after serially diluting and plating on GCK plates. Shown are the averages (±SD) of 2-3 independent experiments. (**B**) MS11Opa$_{CEA}$ were cultured in the absence and presence of the NF-κB inhibitor Bay11-7082 (3 μM) in CMRL-1066 media containing 5% FBS for 24 h. Optical density at 650 nm was measured at 0, 6, 12, and 24 h. Shown are the averages (±SD) of 3 independent experiments. There are no statistically significant differences by pair student's *t*-test.

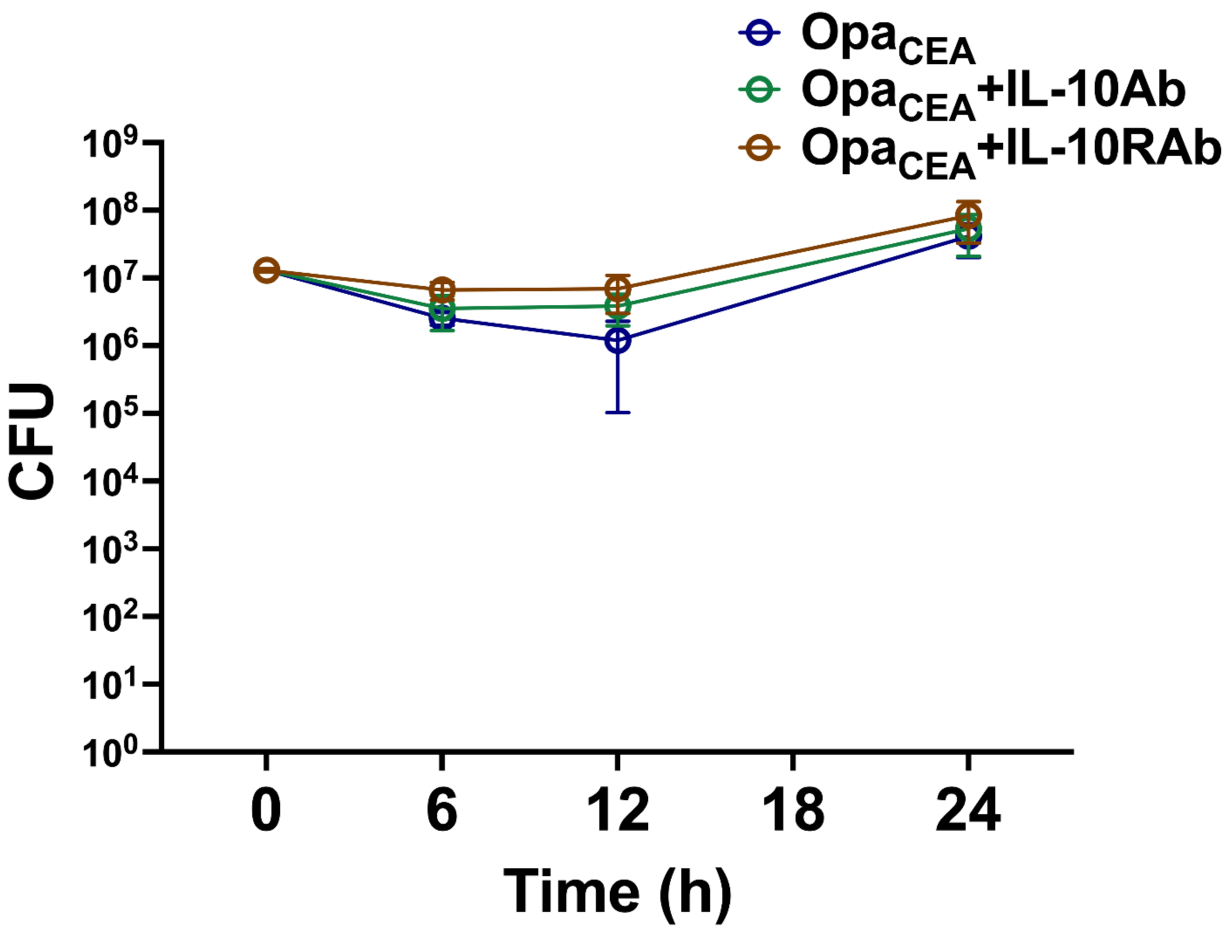

Supplementary Figure 4. IL-10 neutralization and IL-10 receptor α-blocking antibodies do not affect GC viability. MS11Opa$_{CEA}$ were cultured in CMRL-1066 media containing 5% FBS for 0, 6, 12, and 24 h in the absence or presence of the IL-10 neutralization (10 µg/ml) and IL-10 receptor (IL-10R) α-blocking antibodies (5 µg/ml). Bacteria were enumerated by counting CFU after serially diluting tissue culture media and plating on GCK plates. Shown are the averages (±SD) of 3 independent experiments. There are no statistically significant differences by One-Way ANOVA.

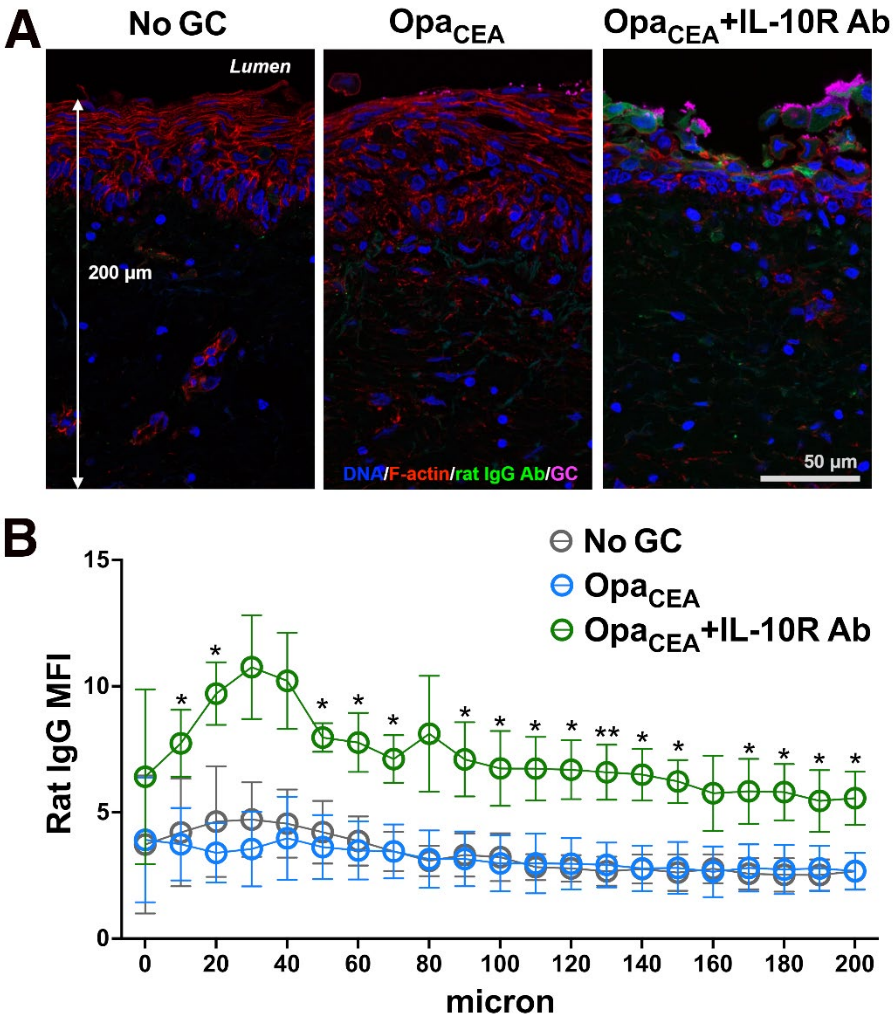

Supplementary Figure 5. IL-10Rα-blocking antibody primarily targets cervical epithelial cells. Human ectocervical tissue explants were incubated without and with MS11Opa$_{CEA}$ (MOI~10) in the absence and presence of rat IgG anti-human IL-10Rα antibody (5 µg/ml) for 24 h and cryopreserved. Tissue sections were stained with anti-rat-IgG, GC-specific antibodies, phalloidin, and Hoechst and imaged by CFM. (**A**) Representative images of the ectocervical tissues. Scale bar, 50 µm. (**B**) Quantification of rat IgG MFI from the luminal surface of the ectocervical epithelium to 200 µm depth into the subepithelium. Shown are the average values (±SD) generated from 3-4 ectocervixes and 5-8 randomly taken images per cervix. * p<0.05, ** p<0.01 by Two-Way ANOVA.

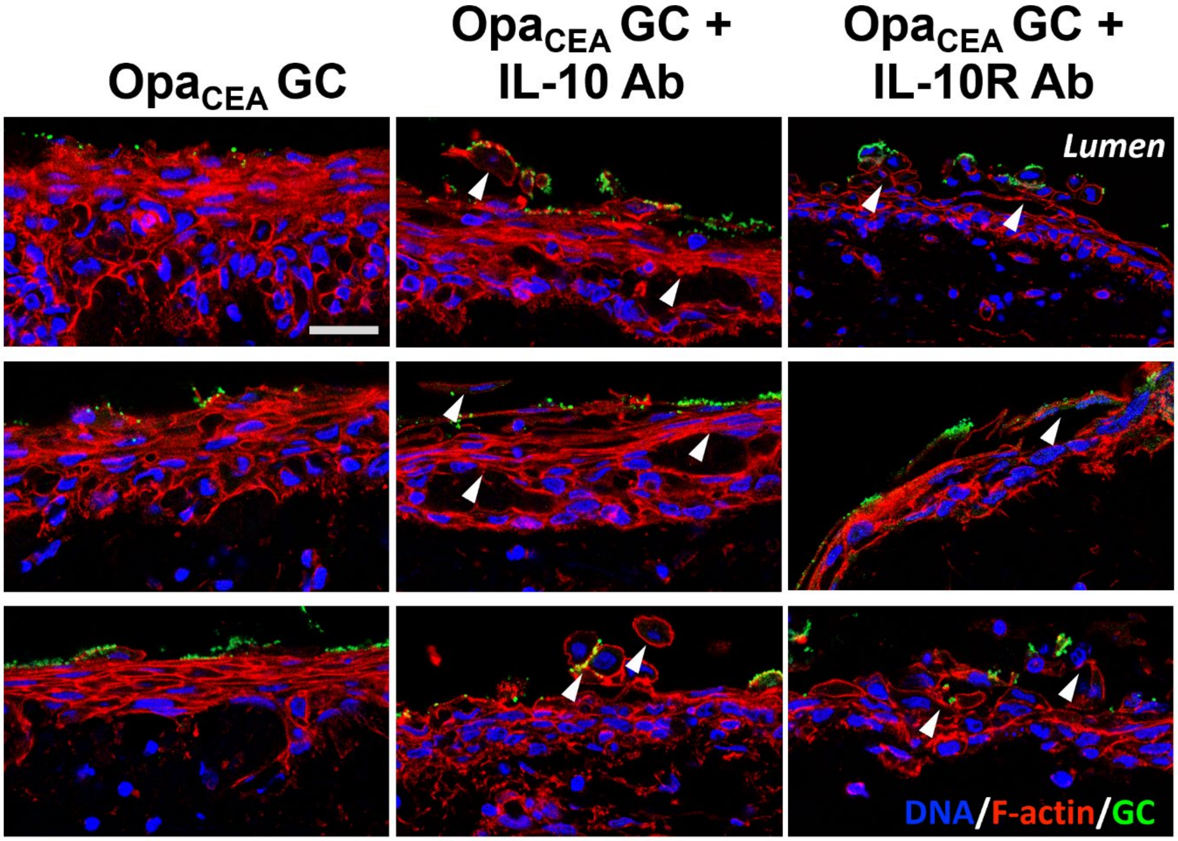

Supplementary Figure 6. IL-10 neutralization and IL-10 receptor-blocking antibodies reduce GC colonization and increase ectocervical epithelial cell shedding. Human cervical tissue explants were incubated with MS11Opa$_{CEA}$ (MOI~10) in the absence and presence of anti-IL-10 (10 µg/ml) or IL-10Rα antibody (5 µg/ml) for 24 h. The tissues were cryopreserved. Ectocervical tissue sections were stained for GC, DNA, and F-actin. Shown are three sets of example images of ectocervical tissues. Arrowhead, shed epithelial cells. Scale bar, 10 µm.
